# Genome-Wide Identification of Dicer-Like, Argonaute, and RNA-Dependent RNA Polymerase Gene Families in *Brassica* Species and Functional Analyses of Their Arabidopsis Homologs in Resistance to *Sclerotinia sclerotiorum*

**DOI:** 10.3389/fpls.2016.01614

**Published:** 2016-10-27

**Authors:** Jia-Yi Cao, You-Ping Xu, Wen Li, Shuang-Sheng Li, Hafizur Rahman, Xin-Zhong Cai

**Affiliations:** ^1^Institute of Biotechnology, College of Agriculture and Biotechnology, Zhejiang UniversityHangzhou, China; ^2^Center of Analysis and Measurement, Zhejiang UniversityHangzhou, China

**Keywords:** *Brassica napus*, RNA silencing, Dicer-like (DCL), Argonaute (AGO), RNA-dependent RNA polymerase (RDR), *Sclerotinia sclerotiorum*, CAMTA3

## Abstract

RNA silencing is an important mechanism to regulate gene expression and antiviral defense in plants. Nevertheless, RNA silencing machinery in the important oil crop *Brassica napus* and function in resistance to the devastating fungal pathogen *Sclerotinia sclerotiorum* are not well-understood. In this study, gene families of RNA silencing machinery in *B. napus* were identified and their role in resistance to *S. sclerotiorum* was revealed. Genome of the allopolyploid species *B. napus* possessed 8 *Dicer-like* (*DCL*), 27 *Argonaute* (*AGO*), and 16 *RNA-dependent RNA polymerase* (*RDR*) genes, which included almost all copies from its progenitor species *B. rapa* and *B. oleracea* and three extra copies of *RDR5* genes, indicating that the *RDR5* group in *B. napus* appears to have undergone further expansion through duplication during evolution. Moreover, compared with Arabidopsis, some *AGO* and *RDR* genes such as *AGO1, AGO4, AGO9*, and *RDR5* had significantly expanded in these *Brassica* species. Twenty-one out of 51 *DCL, AGO*, and *RDR* genes were predicted to contain calmodulin-binding transcription activators (CAMTA)-binding site (CGCG box). *S. sclerotiorum* inoculation strongly induced the expression of *BnCAMTA3* genes while significantly suppressed that of some CGCG-containing RNA silencing component genes, suggesting that RNA silencing machinery might be targeted by CAMTA3. Furthermore, Arabidopsis mutant analyses demonstrated that *dcl4-2, ago9-1, rdr1-1, rdr6-11*, and *rdr6-15* mutants were more susceptible to *S. sclerotiorum*, while *dcl1-9* was more resistant. Our results reveal the importance of RNA silencing in plant resistance to *S. sclerotiorum* and imply a new mechanism of CAMTA function as well as RNA silencing regulation.

## Introduction

RNA silencing refers to a variety of mechanisms whereby a small RNA molecule interferes with a given nucleotide sequence. It was first discovered in plants and occurs widely in eukaryotic organisms (Tijsterman et al., 2002; Ullu et al., 2004). In plants, RNA silencing is triggered by double-stranded RNA (dsRNA) that gives rise to small RNAs known as microRNAs (miRNAs) or small-interfering RNAs (siRNAs). Generation and function of these small RNAs depend on three key protein families, Dicer-like proteins (DCLs), Argonautes (AGOs), and RNA-dependent RNA polymerases (RDRs; Baulcombe, 2004). A whole RNA silencing process comprises three stages: initiation, maintenance, and signal amplification. DCLs undergo RNase III-type activities to process complementary double-strand RNAs into small RNAs with 21–26 nucleotides in length (Carmell and Hannon, 2004). These small RNAs are then incorporated into AGO-containing RNA-induced silencing complexes (RISCs) to serve as the sequence specificity in RNA degradation, translational inhibition, or heterochromatin formation (Bologna and Voinnet, 2014). At the signal amplification stage, RDR enzymes are responsible for synthesis of dsRNAs from ssRNA templates to initiate a new round of RNA silencing (Sijen et al., 2001).

DCL, AGO, and RDR are key components of RNA silencing machinery. DCL proteins are key components in small RNA biogenesis. They are characterized by the presence of six domains: DEAD, helicase-C, DUF283, PAZ, RNase III, and dsRNA-binding motif (DSRM; Margis et al., 2006). DCL consists of a small gene family in higher plants, insects, protozoa, and some fungi, whereas only one Dicer protein exists in vertebrates, nematodes, *Schizosaccharomyces pombe*, and green alga *Chlamydomonas reinhardtii* (Liu et al., 2009). AGO proteins are core factors of the RISC that guide small RNAs to their targets by sequence complementarity, which results in target mRNA cleavage, translational repression, or chromatin modification (Hannon, 2002; Moazed, 2009). AGOs are large proteins (~90–100 kDa) consisting of several characteristic functional domains including DUF1785, PAZ, MID, and PIWI (Hutvagner and Simard, 2008). RDRs enhance the potency of RNAi by amplifying the aberrant RNA population. It is defined by the presence of a conserved RNA-dependent RNA polymerase catalytic domain and is required for initiation and amplification of the silencing signal (Schiebel et al., 1998). Multiple copies of AGO and RDR genes are known to exist in both plants and animals. Members of these gene families play different roles in RNA silencing. For example, the *Arabidopsis thaliana* genome contains four DCL proteins (DCL1–DCL4) that specifically produce different types and sizes of small RNAs (Bologna and Voinnet, 2014). The role of DCL1 is to extract a single small RNA duplex out of a RNA loop called the pri-miRNA. This gives rise to a miRNA, generally 21 nucleotides long, and is typically involved in regulating developmental genes (Parent et al., 2015). DCL2 can produce abundant 22-nt viral siRNAs and shares functional overlap with DCL4 in antivirus defense (Moissiard et al., 2007). DCL3 generates 24-nt repeat-associated siRNAs (ra-siRNAs) and is involved in antiviral defense against DNA viruses (Moissiard and Voinnet, 2006). DCL4 generates 21-nt trans-acting siRNAs (ta-siRNA) and is the primary DCL component of antiviral defense against RNA viruses (Deleris et al., 2006). Likewise, AGO1, the most well-studied plant AGO gene, associates with miRNAs and some siRNAs such as ta-siRNAs to cleave target mRNA and/or inhibit translation (Yu and Wang, 2010). AGO2 protein is involved in antiviral defense by catalyzing viral RNA cleavage in Arabidopsis (Jaubert et al., 2011). AGO10, the closest paralog of AGO1, is functionally redundant with AGO1 in some aspects of development (Lynn et al., 1999) and also functions as a decoy for miR165/166 to prevent the formation of AGO1-miR165/166 complexes and the subsequent repression of HDZIP III gene expression (Zhu et al., 2011). For RDRs, RDR2 converts ssRNAs generated from repetitive DNAs to precursor dsRNAs of ra-siRNAs (Xie et al., 2004), while RDR6 produces the ta-siRNA precursors (Yoshikawa et al., 2005). Gene families encoding these three key components of RNA silencing machinery have been identified only in several plant species such as *A. thaliana, Oryza sativa* (Kapoor et al., 2008), *Zea mays* (Qian et al., 2011), *Solanum lycopersicum* (Bai et al., 2012), *Nicotiana benthamiana* (Nakasugi et al., 2013), *Setaria italica* (Yadav et al., 2015), and *Vitis vinifera* (Zhao et al., 2015). Identification of these families in more plant species will enhance our understanding of RNA silencing.

RNA silencing plays multiple roles in regulating growth and development as well as abiotic and biotic stress responses. In higher plants, RNA silencing functions as an antiviral defense through the action of DCL, AGO, and RDR proteins (Ding and Voinnet, 2007). The importance of RNA silencing in plant viral defense is manifested by the fact that it has elicited counter defense measures from the viral pathogens to overcome it. Plant viruses have evolved various viral RNA silencing repressors (VSR) to counteract this defense mechanism by targeting different RNA silencing pathway components (Csorba et al., 2015). Apart from viral defense, evidence accumulates for RNA silencing to play a role in plant interactions with bacterial pathogens (Voinnet, 2008). The first example is a miRNA from Arabidopsis that contributes to basal defense against *Pseudomonas syringae* by regulating auxin signaling (Navarro et al., 2006). Similar to viruses, the bacteria has also developed mechanisms to suppress RNA silencing in order to cause disease (Navarro et al., 2008). Recently, through the use of mutants for key components of RNA silencing or functional analyses of miRNAs in plant defense, the potential role of RNA silencing in plant defense against fungal pathogens has been revealed. These fungi include *Verticillium dahliae* (Ellendorff et al., 2009), *Verticillium longisporum* (Shen et al., 2014), *Magnaporthe oryzae* (Li et al., 2014), and *Botrytis cinerea* (Jin and Wu, 2015).

*Brassica napus* is an allotetraploid and was formed about 7500 years ago by crossing between *B. oleracea* and *B. rapa*, followed by chromosome doubling (Chalhoub et al., 2014). It is one of the most important oil crops, yet few RNAi machinery components have been characterized to date. We have identified the miRNAs involved in the interactions between *B. napus* and *Sclerotinia sclerotiorum*, one of the most devastating fungal pathogens in oil and vegetable crops. Furthermore, we find that Arabidopsis *ago1* and *ago2* mutant plants exhibit enhanced susceptibility to *S. sclerotiorum* (Cao et al., 2016). Our results provide a clue to the important roles of RNA silencing in the interactions between *B. napus* and *S. sclerotiorum*. In this study, taking advantage of the completion of the *B. napus* genome sequencing (Chalhoub et al., 2014), we performed comprehensive bioinformatics analyses to identify DCL, AGO, and RDR gene families that are the three key components of RNA silencing machinery in *B. napus*. Furthermore, we employed mutants to probe their functions in resistance to *S. sclerotiorum*. We revealed the significant difference in RNA silencing machinery composition between *B. napus* and Arabidopsis, demonstrated the important role of RNA silencing in resistance to *S. sclerotiorum* and indicated a possible regulating mechanism of RNA silencing.

## Materials and methods

### Identification of putative *B. napus DCL, AGO*, and *RDR* genes

Protein sequences of Arabidopsis DCLs, AGOs, and RDRs were downloaded from TAIR database (http://www.arabidopsis.org/) and scan for conserved domains were performed using National Center for Biotechnology Information Conserved Domain Database (NCBI-CDD; http://www.ncbi.nlm.nih.gov/Structure/cdd/wrpsb.cgi). All these protein sequences were used as queries to search their orthologs in *B. napus, B. rapa*, and *B. oleracea* genomes using BLASTp program in NCBI database with default settings. All retrieved protein sequences were examined for the presence of conserved domains and redundant sequences were removed. All candidate sequences of *B. napus* were subsequently verified in the GENOSCOPE database (http://www.genoscope.cns.fr/blat-server/cgi-bin/colza/webBlat). The physico-chemical properties of BnDCL, BnAGO, and BnRDR proteins were then predicted using ExPASy Compute pI/Mw tool (http://au.expasy.org/tools/pi_tool.html; Bjellqvist et al., 1993).

### Phylogenetic analysis and nomenclature

Multiple alignment of DCL, AGO, and RDR protein sequences from *A. thaliana, B. napus, B. rapa*, and *B. oleracea* was performed using MUSCLE program (Edgar, 2004). The phylogenetic trees were then constructed using MEGA 5.0 (Tamura et al., 2011) by Neighbor-Joining (NJ) method following the Jones-Taylor-Thornton (JTT) model. Bootstrap analysis was performed with 1000 replicates to assess statistical support for nodes. The candidate genes were renamed according to the phylogenetic relationship and sequence homologies with corresponding *A. thaliana* homologs. The detail information of the proteins used for phylogenetic tree construction was listed in Table 1 and Table S1.

**Table 1:** **List of *B. napus DCL, AGO*, and *RDR* genes**.

**Gene no**.	**Gene name**	**Accession no**.	**CDS length (bp)**	**Predicted protein**			**No. of introns**	**Genomic location**
				**Length (aa)**	**pI**	**Mw (kDa)**		
***DCLs***								
1	*BnDCL1A*	BnaA10g00800D	5439	1812	5.97	202.15	17	chrA10:390521.398004
2	*BnDCL1C*	BnaC05g00860D	5430	1809	6.01	201.54	19	chrC05:435258.442575
3	*BnDCL2A*	BnaA05g32540D	4167	1388	6.77	156.70	21	chrA05:22308269.22314738
4	*BnDCL2C*	BnaC05g47910D	4170	1389	7.65	156.94	21	chrC05:42659693.42666090
5	*BnDCL3A*	XP_013656716^a^	4572	1523	5.91	141.60	23	A8 NC_027764.1 (14560641.14567368)
6	*BnDCL3C*	BnaC03g54010D	4596	1531	6.04	172.25	24	chrC03:40673425.40680494
7	*BnDCL4A*	BnaA10g15080D	4929	1642	6.22	185.01	24	chrA10:11827194.11836581
8	*BnDCL4C*	BnaC09g37430D	4926	1641	6.22	184.48	24	chrC09:40709757.40719200
***AGOs***								
1	*BnAGO1A1*	BnaA08g03260D	3135	1044	9.40	115.85	22	chrA08:2681072.2686918
2	*BnAGO1A2*	BnaA05g17460D	3261	1086	9.38	120.33	22	chrA05:12290150.12296889
3	*BnAGO1C1*	BnaC08g46720D	3159	1052	9.45	116.78	20	chrC08_random:953598.958888
4	*BnAGO1C2*	BnaC05g25730D	2943	980	9.17	109.15	22	chrC05:21116236.21122145
5	*BnAGO2A*	BnaA09g25290D	3072	1023	9.66	113.91	3	chrA09:18324937.18328648
6	*BnAGO2C*	BnaCnng68320D	2664	887	9.44	100.70	2	chrCnn_random:67905947.67909115
7	*BnAGO3A*	BnaA05g14760D	3033	1010	9.51	112.74	1	chrA05:9228612.9232112
8	*BnAGO3C*	BnaC06g41790D	3108	1035	9.54	114.35	1	chrC06_random:1066401.1069740
9	*BnAGO4A1*	BnaA04g15560D	2769	922	8.91	103.06	21	chrA04:12884285.12890171
10	*BnAGO4A2*	BnaA07g13010D	2772	923	8.82	103.36	21	chrA07:11653377.11659092
11	*BnAGO4C1*	BnaC04g38560D	2769	922	8.87	103.11	21	chrC04:39739228.39745117
12	*BnAGO4C2*	BnaC04g54830D	2772	923	8.87	103.36	21	chrC04_random:2230442.2236093
13	*BnAGO5A*	BnaA07g13430D	2874	957	9.52	106.71	19	chrA07:11907998.11912994
14	*BnAGO5C*	BnaC04g16450D	2859	952	9.62	106.03	20	chrC04:14487678.14492897
15	*BnAGO6A*	BnaA03g15180D	2604	867	9.03	97.20	21	chrA03:7005357.7010304
16	*BnAGO6C*	BnaC03g18310D	2604	867	8.99	97.37	21	chrC03:9391663.9396398
17	*BnAGO7A*	BnaA07g24280D	2955	984	9.37	112.47	2	chrA07:18160385.18163845
18	*BnAGO7C1*	BnaC02g19190D	2931	976	9.32	111.58	2	chrC02:15451981.15455314
19	*BnAGO7C2*	BnaC06g43420D	2700	899	9.38	102.67	5	chrC06_random:2865213.2868659
20	*BnAGO8A*	BnaA02g05290D	2721	906	9.31	101.34	20	chrA02:2403187.2408757
21	*BnAGO9A1*	BnaA10g14450D	2715	904	9.31	102.04	21	chrA10:11492941.11497567
22	*BnAGO9A2*	BnaA10g14440D	2748	915	9.38	102.52	20	chrA10:11481378.11486279
23	*BnAGO9C1*	BnaCnng35060D	2721	906	9.42	102.57	21	chrCnn_random:33265084.33269730
24	*BnAGO9C2*	BnaC09g36780D	2763	920	9.20	103.80	23	chrC09:40119310.40124830
25	*BnAGO9C3*	BnaC09g36860D	2649	882	9.23	99.37	19	chrC09:40226286.40231475
26	*BnAGO10A*	BnaA06g36540D	2928	975	9.38	109.27	16	chrA06:23915363.23920283
27	*BnAGO10C*	BnaC07g17330D	2946	981	9.38	109.75	16	chrC07:23533982.23539648
***RDRs***								
1	*BnRDR1A*	BnaA06g09600D	3330	1109	7.50	126.63	2	chrA06:5134289.5137990
2	*BnRDR1C1*	XP_013669643^a^	3207	1068	5.93	84.40	3	C1 NC_027767.1 (42513981.42517438)
3	*BnRDR1C2*	BnaC05g10980D	3282	1093	6.62	124.83	3	chrC05:6358607.6362657
4	*BnRDR2A*	BnaA09g22040D	3390	1129	6.15	128.25	3	chrA09:14659131.14663050
5	*BnRDR2C*	BnaCnng57100D	3378	1125	6.06	127.52	3	chrCnn_random:56892147.56896339
6	*BnRDR3A*	BnaA09g43930D	3003	1000	8.34	113.41	16	chrA09:30278290.30284646
7	*BnRDR3C*	BnaC08g36490D	3000	999	8.05	113.19	16	chrC08:33743646.33750434
8	*BnRDR4A*	BnaA10g12910D	2982	993	8.58	112.64	17	chrA10:10516020.10520784
9	*BnRDR5A1*	BnaA07g00800D	2928	975	7.13	110.64	19	chrA07:581670.586372
10	*BnRDR5A2*	XP_013652716^a^	2853	951	6.32	84.22	16	A7 NC_027763.1 (1493563.1498604, complement)
11	*BnRDR5A3*	BnaA07g00770D	2757	918	6.08	103.58	16	chrA07:561541.565534
12	*BnRDR5C1*	BnaC07g01150D	2934	977	6.10	110.60	17	chrC07:1764972.1769964
13	*BnRDR5C2*	BnaC07g01170D	2877	958	6.61	108.36	16	chrC07:1805711.1810421
14	*BnRDR5C3*	BnaC07g01120D	2586	861	5.65	97.84	16	chrC07:1738692.1746427
15	*BnRDR6A*	XP_013655642^a^	3597	1198	6.93	137.07	1	A8 NC_027764.1 (3130135.3141222, complement)
16	*BnRDR6C*	XP_013720258^a^	3597	1198	6.78	137.03	1	Unplaced scaffold NW_013650343.1 (584631.589152)

a The noted sequences were from the NCBI BioProject: PRJNA293435, while all the others were from the NCBI BioProject: PRJEB5043.

### Exon-intron structure analysis and promoter *cis*-acting element prediction

The exon-intron organization of *BnDCLs, BnAGOs*, and *BnRDRs* genes were determined using the online GSDS1.0 program (http://gsds.cbi.pku.edu.cn/) by comparing their full-length coding sequences (CDS) with their corresponding genomic sequences downloaded from the GENOSCOPE database. The upstream sequences (1.5 kb) of *BnDCL, BnAGO*, and *BnRDR* genes were searched for the presence of potential *cis*-acting elements using PLACE database (http://www.dna.affrc.go.jp/PLACE/signalup.html; Higo et al., 1999).

### Plant materials and inoculation analyses

The Arabidopsis *dcl, ago*, and *rdr* mutants were provided by Prof. Shou-Wei Ding (Department of Plant Pathology and Microbiology, University of California, Riverside, USA) and Prof. Yi-Jun Qi (Tsinghua-Peking Center for Life Sciences, and School of Life Sciences, Tsinghua University, China). *B. napus* plants were grown in growth cabinets at 25°C under a 16/8 h light/dark photoperiod, while Arabidopsis plants of the wild-type and mutants of RNA-silencing related genes were grown at 23°C with a 12/12 h day/night photoperiod. Fresh sclerotia of *S. sclerotiorum* were cultured at 23°C on potato dextrose agar medium (PDA) to produce mycelia, which were transferred to new PDA plates and grown for 2 days. The PDA plugs containing the advancing edge of *S. sclerotiorum* mycelia were removed to inoculate the plant leaves. For gene expression analyses, leaves were collected at 0, 8, and 16 h post inoculation (hpi) and frozen immediately in liquid nitrogen. Diameter of disease lesions was measured at 24 hpi and statistically analyzed using SPSS (verson19.0) by Student's *t*-test (*p* < 0.05). For disease resistance evaluation, at least 10 plants for each genotype were examined and the experiments were conducted three times independently.

### Real-time quantitative PCR

Total RNA was extracted using Trizol reagent (Invitrogen, CA, USA) following the manufacturer's procedure. Real-time quantitative PCR (RT-qPCR) was carried out using SYBR Premix Ex Taq (TakaRa, China) on StepOne Real-Time PCR System (ABI, USA).The RT-qPCR analyses were conducted three times, with three replicates for each gene and the relative fold changes were calculated using the 2^−ΔΔCt^ method as described (Zhao et al., 2013). A *B. napus* elongation factor gene was used as the reference gene and primers used for RT-qPCR are listed in Table S2. Significance of the differences between mean values was determined with Student's *t*-test (*p* < 0.05).

## Results

### Genome-wide identification of *DCL, AGO*, and *RDR* genes in *B. napus*

A BLASTp search was conducted against *B. napus* genome in NCBI database using well-characterized Arabidopsis AGO, DCL, and RDR protein sequences as query sequences. The retrieved sequences were further analyzed for domain composition. Finally, 8 *DCL*, 27 *AGO*, and 16 *RDR* genes were identified in *B. napus* genome (Table 1). Compared with *A. thaliana* which contains 4 *DCL*, 10 *AGO*, and 6 *RDR* genes (Table S1); the members for each gene family in *B. napus* expanded by two times or more (Table 1). To compare the composition of these RNA silencing machinery genes between *B. napus* and its progenitor species *B. rapa* and *B. oleracea*, similar BLASTp and domain identification analyses were performed for these two genomes. The results showed that *B. rapa* genome contained 4 *DCL*, 13 *AGO*, and 6 *RDR* genes while *B. oleracea* genome carried 4 *DCL*, 14 *AGO*, and 7 *RDR* genes (Table S1). Comparison analysis indicated that *B. napus* genome possessed all copies of *DCL, AGO*, and *RDR* genes from the two progenitor species and contained three extra copies of *RDR* genes (Table 1 and Table S1).

### Classification of *B. napus DCL, AGO*, and *RDR* genes based on phylogenetic analysis

**Figure 1:**
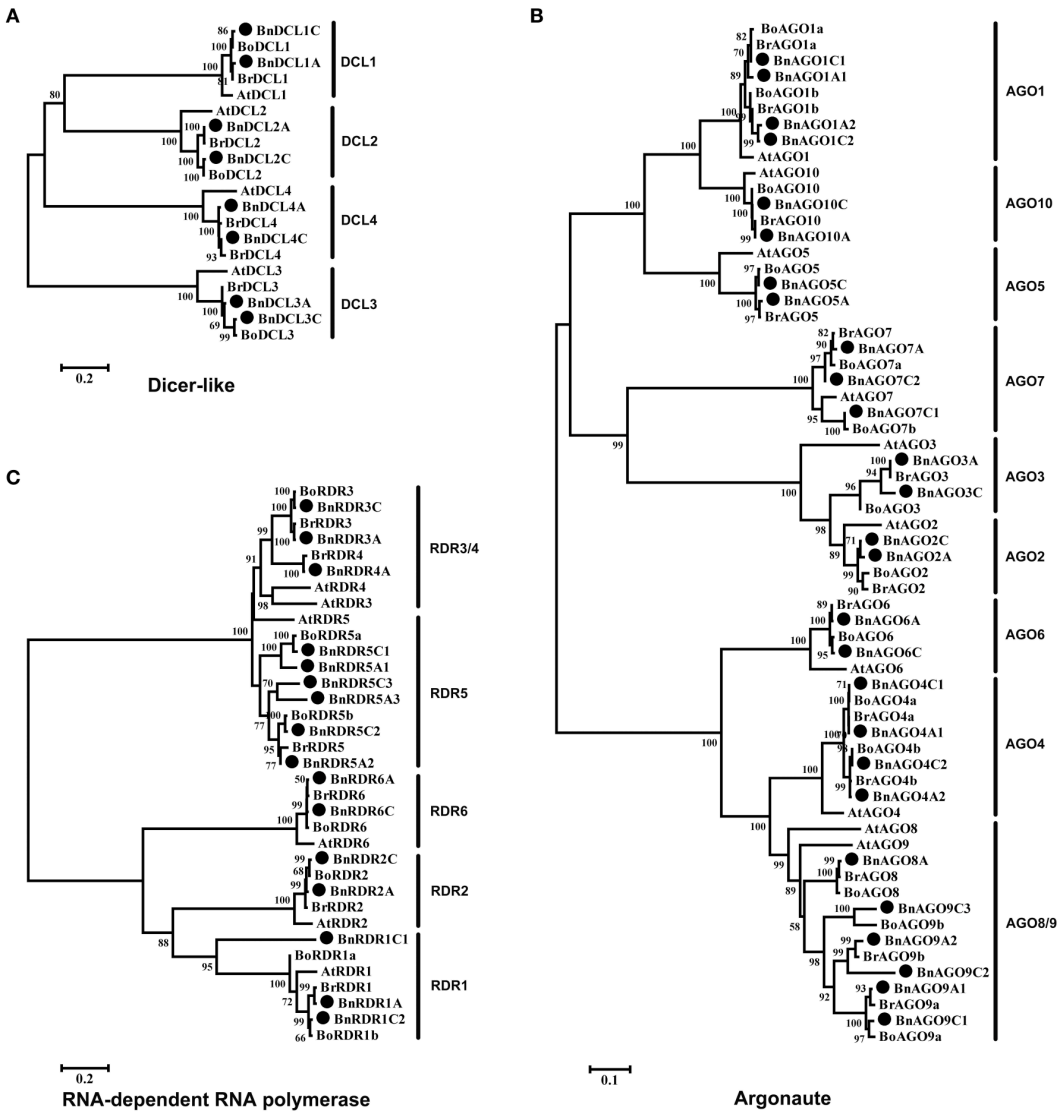
**Phylogenetic trees of *B. napus* and *A. thaliana* Dicer-like (A), Argonaute (B), and RNA-dependent RNA polymerse (C) protein sequences**. The trees were created by MEGA 5.0 software using Neighbor-Joining (NJ) methods with bootstrap of 1000. *B. napus* proteins are indicated with a black filled circle before the protein names.

In order to examine the phylogenetic relationship of DCL, AGO, and RDR families, we constructed unrooted phylogenetic trees of all BnDCL, BnAGO, and BnRDR protein sequences along with their *A. thaliana, B. rapa*, and *B. oleracea* homologs (Figure 1). The 8 BnDCLs were obviously divided into four groups as reported for AtDCLs. Each group comprised two members with one contributed by each subgenome (A and C; Figure 1A). Coincidently, *B. rapa* and *B. oleracea* contained 4 DCLs with one member for each group (Figure 1A). According to the phylogenetic relationship and sequence homology with AtDCLs, the 8 BnDCLs were named as BnDCL1A, BnDCL1C, BnDCL2A, BnDCL2C, BnDCL3A, BnDCL3C, BnDCL4A, and BnDCL4C in accordance with their genomic localization (in A or C). Besides, BnDCLs showed high sequence similarities (from 78.7 to 87.6% for each group) to their Arabidopsis counterparts (Table S3).

Based on the NJ phylogenetic tree and the protein sequence homologies with AtAGOs, the BnAGOs family consisted of 4 AGO1s (BnAGO1A1, BnAGO1A2, BnAGO1C1, and BnAGO1C2), 2 AGO2s (BnAGO2A and BnAGO2C), 2 AGO3s (BnAGO3A and BnAGO3C), 4 AGO4s (BnAGO4A1, BnAGO4A2, BnAGO4C1, and BnAGO4C2), 2 AGO5s (BnAGO5A and BnAGO5C), 2 AGO6s (BnAGO6A and BnAGO6C), 3 AGO7s (BnAGO7A, BnAGO7C1, and BnAGO7C2), 1 AGO8 (BnAGO8A), 5 AGO9s (BnAGO9A1, BnAGO9A2, BnAGO9C1, BnAGO9C2, and BnAGO9C3) and 2 AGO10s (BnAGO10A and BnAGO10C). The total *B. napus* AGO copies in each subgroup were corresponding to that in the two progenitors *B. rapa* and *B. oleracea* except one extra AGO9 (BnAGO9C3) and one less AGO8 (Figure 1B). Notably, an uneven number of AGO gene copies from these three *Brassica* species was observed. Genomes of *B. rapa* and *B. oleracea* comprised two copies of *AGO1, AGO4*, and *AGO9* genes, which was identical to the subgenomes A and C of *B. napus* except that the subgenome C of *B. napus* contained an extra copy of *AGO9* (BnAGO9C3). In addition, *B. oleracea* genome and *B. napus* subgenome C possessed two copies of *AGO7* genes. Thus, copy numbers of these *AGO* genes were higher in these *Brassica* species than in *A. thaliana*. Instead, *B. napus* subgenome C did not contained any *AGO8* gene. Besides the exceptions herein described, the number (only one) of the remaining AGOs in genomes of *B. rapa* and *B. oleracea* and subgenomes A and C of *B. napus* was identical to *A. thaliana*. On the other hand, the distribution of gene members of AGO groups was generally even in the A and C subgenomes of *B. napus* except AGO7, AGO8, and AGO9 (Figure 1B and Table 1). Additionally, sequence similarity between BnAGOs and Arabidopsis homologs was generally high, ranging from 60.8 to 92.7%, while the similarity among sequences of the same group of BnAGOs was 87.9–91.1% for AGO1, 86.7% for AGO2, 90.0% for AGO3, 96.8–99.6% for AGO4, 94.5% for AGO5, 98.6% for AGO6, 76.5–91.0% for AGO7, 72.4–97.3% for AGO9 and 96.1% for AGO10 (Table S3).

Like DCLs and AGOs, RDRs in *B. napus* were named after the Arabidopsis homologs which were the phylogenetically closest in the NJ tree and showed the highest protein sequence homologies. Consequently, *B. napus* geneome comprised 3 RDR1s (BnRDR1A, BnRDR1C1, and BnRDR1C2), 2 RDR2s (BnRDR2A and BnRDR2C), 2 RDR3s (BnRDR3A and BnRDR3C), 1 RDR4 (RDR4A), 6 RDR5s (BnRDR5A1, BnRDR5A2, BnRDR5A3, BnRDR5C1, BnRDR5C2, and BnRDR5C3) and 2 BnRDR6s (BnRDR6A and BnRDR6C; Figure 1C). It is noteworthy that *B. napus* genome possessed 6 *RDR5* genes, 3 in each subgenomes (A and C), which is more than that in genomes of the progenitors *B. rapa* (1) and *B. oleracea* (2), indicating the further multiplication of *RDR5* genes in *B. napus* genome during evolution. Besides, *B. oleracea* genome and *B. napus* subgenome C carried 2 *RDR1* genes but no any *RDR4* gene. Thus, composition of these RDRs in the three *Brassica* genomes was distinct from Arabidopsis. Furthermore, as observed for BnAGO family, members of RDR1 and RDR4 were unequally distributed in the A and C subgenomes of *B. napus*. In addition, protein sequence similarity between BnRDRs and AtRDRs was generally high, ranging from 63.1 to 90.4%, while the similarity within the same group of BnRDRs was 61.5–95.2% for RDR1, 98% for RDR2, 97.6% for RDR3, 70.2–99.3% for RDR5 and 99.4% for RDR6 (Table S3).

Collectively, genome of the allopolyploid species *B. napus* possesses almost all copies of *DCL, AGO*, and *RDR* genes from its progenitor species *B. rapa* and the *RDR5* group in *B. napus* appears to have undergone further expansion through duplication during evolution. Furthermore, compared with Arabidopsis, some *AGO* and *RDR* genes such as *AGO1, AGO4, AGO9*, and *RDR5* have significantly expanded in these *Brassica* species.

### Physico-chemical properties and domain composition of *B. napus* DCL, AGO, and RDR proteins

**Figure 2:**
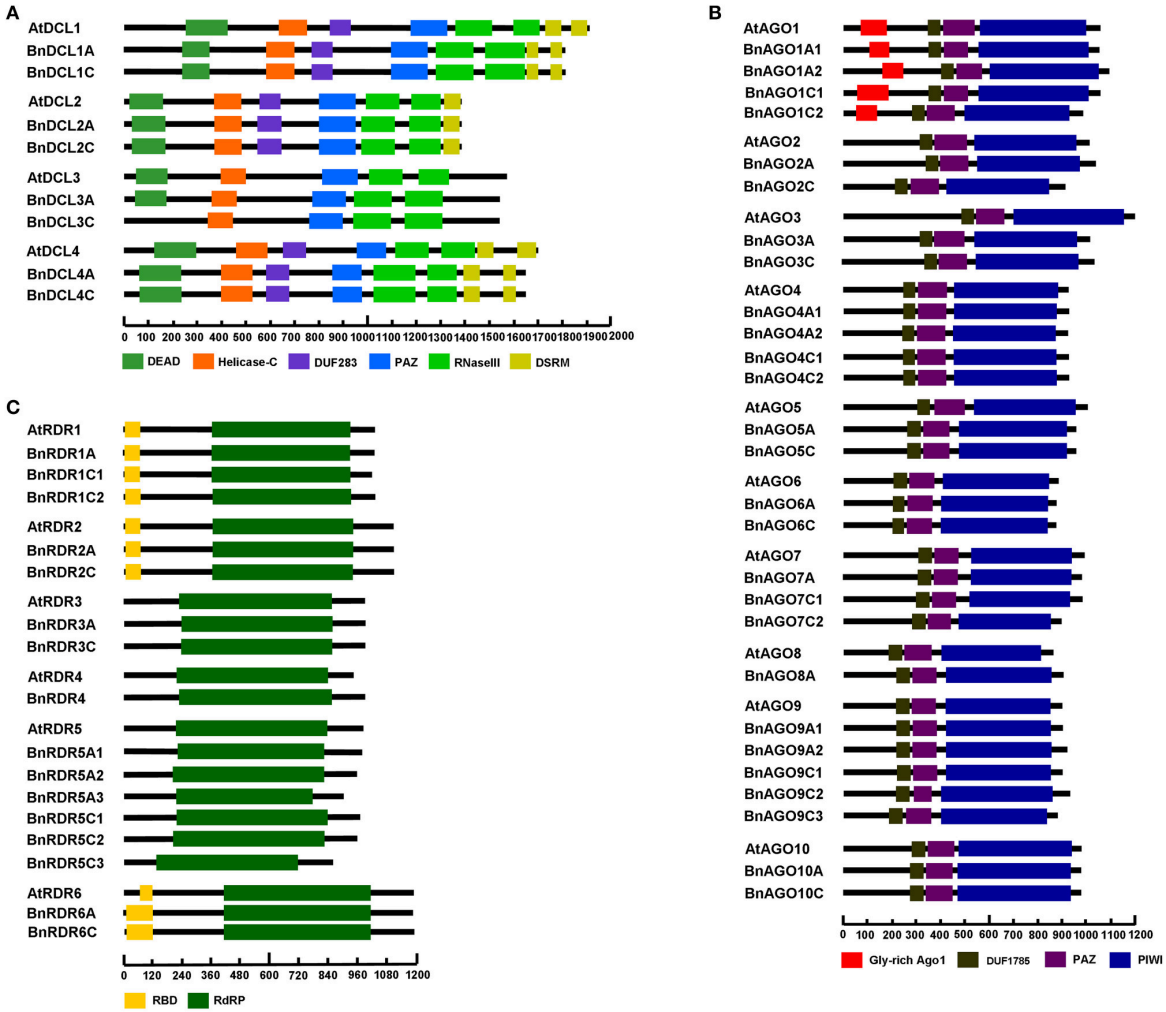
**Domain composition of *B. napus* and *A. thaliana* DCL (A), AGO (B), and RDR (C) protein sequences**. Domains are indicated as boxes in various colors. The diagrams were drawn to scale.

Physico-chemical properties and domain composition of *B. napus* DCL, AGO, and RDR proteins were generally similar to their Arabidopsis counterparts, though some differences were noticed. For DCLs, seven out of eight BnDCL proteins contained DEAD, Helicase-C, Duf283, PAZ, and RNaseIII domains as reported for Arabidopsis DCLs. The remaining one (BnDCL3C) lacked the DEAD domains, which is distinguishable from AtDCL3 in this regard (Figure 2A). Further, comparison of the DEAD domain region of all Arabidopsis and three *Brassica* species revealed two deletions of 31 and 9 amino acids (aa), respectively, in BnDCL3C (Figure S1A). The correct sequence of BnDCL3C awaits further experimental confirmation. The gene length was highly similar within groups but considerably varied between groups of BnDCLs. BnDCL1s were the largest (5439 and 5430 bp) followed by BnDCL4s (4929 and 4926 bp) and BnDCL3s (4572 and 4596 bp), while BnDCL2s were the smallest (4167 and 4170 bp; Table 1). This group-wise variation in gene length is also observed in AtDCLs (Table S1).

**Figure 3:**
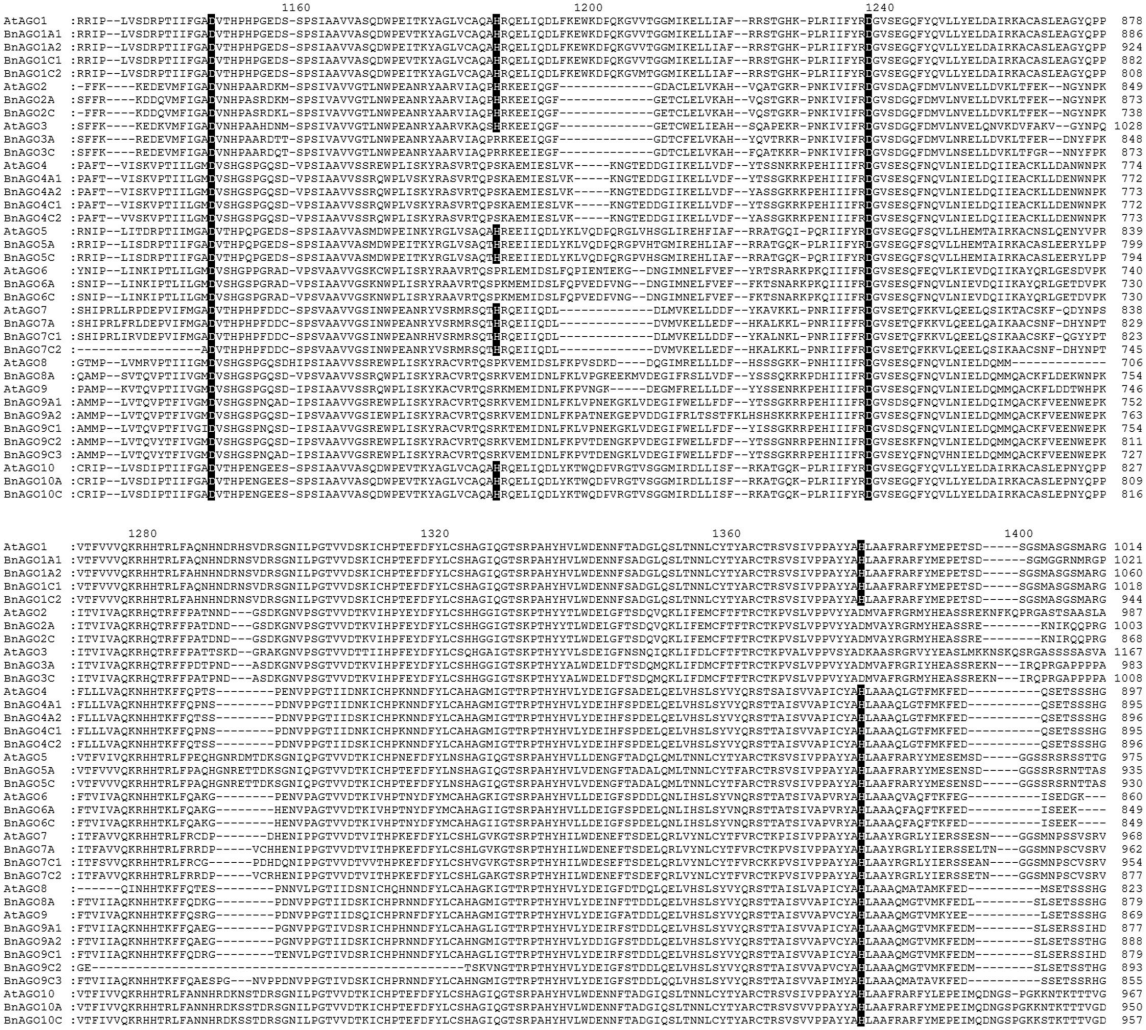
**Alignment of PIWI domains of *B. napus* and *A. thaliana* AGO proteins**. The protein sequences were aligned using MEGA 5.0. The conserved Asp, Asp and His (DDH) triad residues as well as His (H) corresponding to H798 of Arabidopsis AGO1 are shaded black. Amino acid positions corresponding to each protein are indicated at the end of each line.

The domain composition of BnAGOs was identical to that of AtAGOs. All BnAGOs possessed DUF1785, PAZ, and PIWI domains. Besides, all BnAGO1 proteins contained an additional Gly-rich Ago1 domain (Figure 2B). Furthermore, we examined BnAGOs for presence of the four key active residues (DDH/H) in the PIWI domain that are responsible for the endonuclease property of AGO proteins involved in RNAi. The PIWI domain sequence alignment revealed that all 11 members of groups BnAGO1s (4), BnAGO5s (2), BnAGO7s (3), and BnAGO10s (2) possessed the four key active residues, suggesting that they might have endonuclease activity (Figure 3). The remaining BnAGOs belonging to AGO2, AGO3, AGO4, AGO6, AGO8, and AGO9 groups contained substitutions of the two H residues, which is similar to their Arabidopsis AGO counterparts. Furthermore, the length of the *BnAGOs* CDS varied from 2604 bp for *BnAGO6* to 3261 bp for *BnAGO1A2*, potentially encoding 867 and 1086 amino acids, respectively (Table 1). All BnAGOs encode for ~100 kDa basic proteins with a pI ranging from 8.82 to 9.66. The physico-chemical properties of BnAGOs were generally similar to AtAGOs and conserved within all AGO groups.

All the 16 BnRDR proteins carried an RdRP domain (Figure 2C). Additionally, members of BnRDR1, BnRDR2, and BnRDR6 groups bore a RBD domain as observed in the same groups of AtRDRs (Figure 2C). Furthermore, we examined BnRDRs for presence of the catalytic motif in the RdRP domain. The sequence alignment demonstrated that all members of BnRDR1, BnRDR2, and BnRDR6 groups shared a common DLDGD motif in the catalytic domain, while all members of BnRDR3 and five out of six BnRDR5 proteins contained DFDGD motif. The exception was BnRDR5A3, which did not contain DFD in the characteristic catalytic motif, which requires further experimental confirmation (Figure S1B). These data indicated that BnRDR1, BnRDR2, and BnRDR6 proteins belong to RDRα class, while the BnRDR3 and BnRDR5 proteins are members of RDR_γ_ class. This is similar to what has been observed for AtRDRs. Additionally, the length of BnRDR proteins varied from 861 to 1198 aa (Table 1). Different groups of RDRs exhibited diverse pI-values; the pI-value of BnRDR3s and BnRDR4 was higher than 8.0, while that of the majority of the other BnRDRs was lower than 7.0 (Table 1).

Comparison of the protein pI-value indicated that AGOs are obviously basic proteins with a pI higher than 8.8, DCLs are generally acidic proteins with a pI lower than 6.8 except BnDCL2C, while RDRs may be acidic or basic with a pI ranging from 5.7 to 8.6 (Table 1).

### Exon-intron organization of *B. napus DCL, AGO*, and *RDR* genes

The exon-intron structure of *DCL, AGO*, and *RDR* genes was examined to gain more insights into their possible structural evolution. Our results for all three gene families showed that intron number was generally conserved in members of the same groups while varied significantly in different groups of the same family. For *BnDCL* genes, the intron number varied from 17 to 24 and *BnDCL3* and *BnDCL4* groups contained 3~7 more introns than the remaining two groups (Table 1 and Figure S2A). In the case of *BnAGOs*, considerable variations in intron number were observed. *BnAGO* groups 1, 4, 5, 6, 8, 9, and 10 comprised similar number of introns ranging from 16 to 23, while *BnAGO* groups 2, 3, and 7 possessed only 1~5 introns, which were dramatically less than other *BnAGO* groups (Table 1 and Figure S2B). Similar to *BnAGOs*, remarkable intron number variation also occurred in *BnRDRs. BnRDR1, BnRDR2*, and *BnRDR6* groups contained as few as 1~3 introns, while *BnRDR3, BnRDR4*, and *BnRDR5* groups carried as many as 16~19 introns (Table 1 and Figure S2C). These group-dependent exon-intron structures were conserved for genes from both *B. napus* and Arabidopsis. The observation that exon-intron structure of *DCL, AGO*, and *RDR* genes was highly similar within members of the same groups but significantly divergent across different groups of the same family suggests that these gene families especially AGO and RDR families have undergone frequent gene duplication and recombination throughout evolution.

### Prediction of *cis*-acting elements in promoter of *B. napus DCL, AGO*, and *RDR* genes

**Figure 4:**
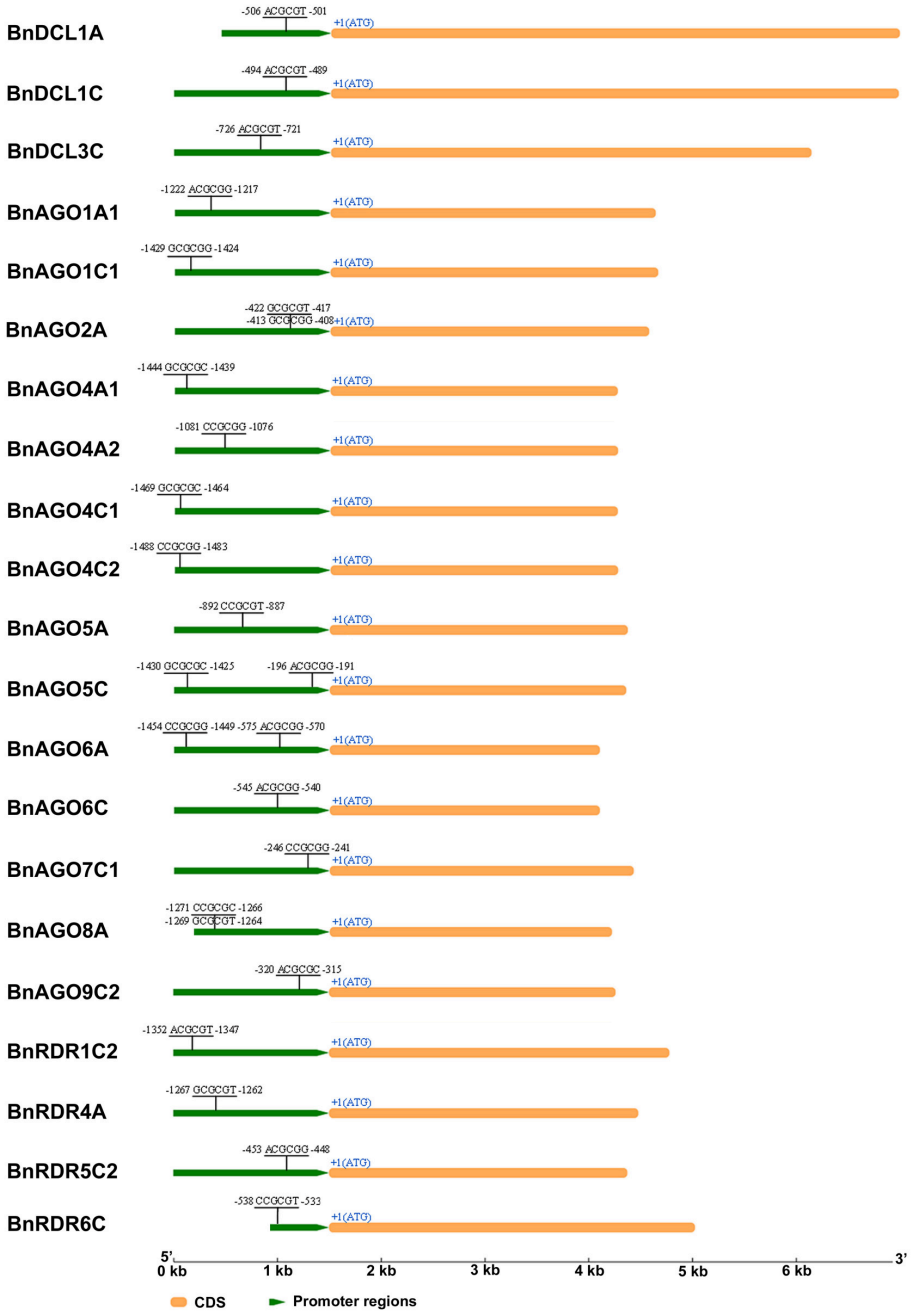
**CGCG *cis*-acting elements of *B. napus DCL*, *AGO*, and *RDR* genes in their promoter regions**. Elements were predicted in 1.5 kb regions upstream from the first ATG. CGCG box sequences and relative positions to the first ATG are shown. The diagrams were drawn to scale.

The 1.5 kb sequences upstream of the translation initiation codon of *BnDCL, BnAGO*, and *BnRDR* genes were retrieved and searched for *cis*-acting elements using PLACE database. This revealed the presence of various *cis*-acting elements related to phytohormone response, abiotic stress and defense response in *BnDCL, BnAGO*, and *BnRDR* genes (Table S4). All of these genes contained at least three *cis*-acting elements directly related to defense response, including ASF1MOTIFCAMV (S000024), GT1CONSENSUS (S000198), SEBFCONSSTPR10A (S000391), WBOXATNPR1 (S000390), and WRKY71OS (S000447). Interestingly, 21 out of these 51 genes possessed a 6-bp CGCG element (A/C/G) CGCG (C/G/T; Figure 4 and Table S4), with *BnAGO2A, BnAGO5C, BnAGO6A*, and *BnAGO8A* containing two copies of these *cis*-elements. It has been revealed that calmodulin-binding transcription activators (CAMTAs) contribute to plant defense responses by binding to CGCG *cis*-elements of the target gene promoters and thereby regulating their expression (Yang and Poovaiah, 2002; Du et al., 2009; Nie et al., 2012). Thus, intriguingly, our result predicts that RNA silencing might be regulated by CAMTAs.

### Expression analyses implied that *CAMTA3* might mediate regulation of *B. napus DCL, AGO*, and *RDR* gene expression in response to pathogen inoculation

**Figure 5:**
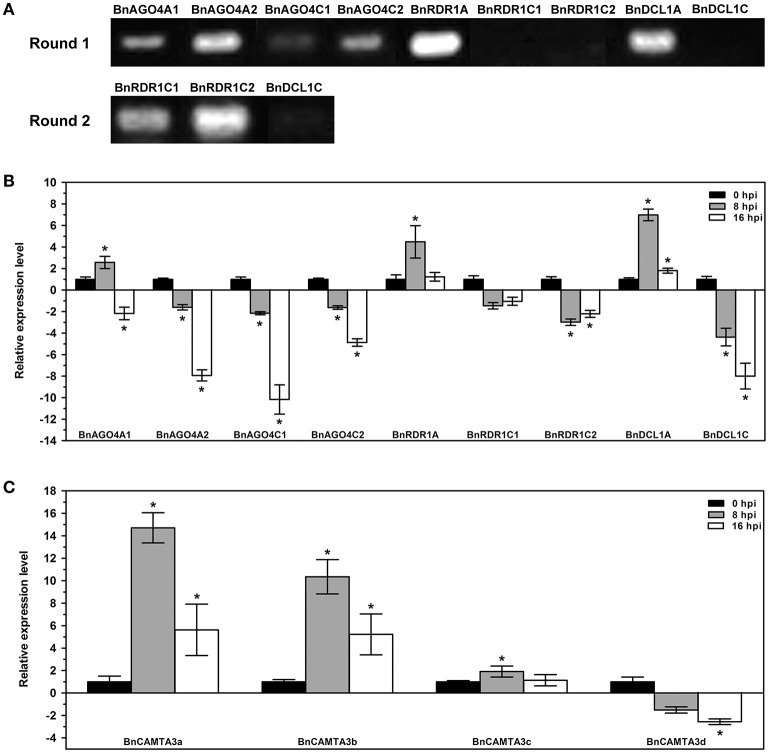
**Expression profiles of *B. napus AGO4*, *RDR1*, *DCL1*, and *CAMTA3* genes. (A)** Semi-quantitative RT-PCR examination of the transcript accumulation of *BnAGO4, BnRDR1*, and *BnDCL1* genes constitutively in *B. napus* leaves under normal growth conditions. Round 2 PCRs were conducted by using the products of the first round PCR as templates. **(B,C)** Quantitative real-time PCR analyses for expression profiles of *BnAGO4, BnRDR1*, and *BnDCL1* genes **(B)** and *BnCAMTA3* genes **(C)** in response to *Sclerotinia sclerotiorum* inoculation in *B. napus* leaves. Expression of these genes at 8 and 16 hpi was shown relatively to that at 0 hpi. The experiments were conducted three times, each containing three replicates for all genes. The expression data were statistically analyzed using SPSS software. Significance of the differences between mean values was determined with Student's *t*-test. Error bars represent *SD*, while asterisks (^*^) indicate significant difference at *p* < 0.05.

Owing to the high cDNA sequence identity in the same gene family, design of gene-specific primers for many of *B. napus DCL, AGO*, and *RDR* genes is unfeasible. Therefore, we chose all gene members of *BnAGO4, BnRDR1*, and *BnDCL1* groups, for which ideal gene-specific primers could be designed, as representative to examine the expression pattern of these predicted genes in *B. napus* and their response to *S. sclerotiorum* inoculation. The analysis demonstrated that these genes were differentially expressed in leaves under normal growth conditions (Figure 5A and Figure S3). Six of them including four *BnAGO4s, BnRDR1A*, and *BnDCL1A*, exhibited moderate to high level expression in normal leaves, while the expression of *BnRDR1C1, BnRDR1C2*, and *BnDCL1C* was only detected when a second round PCR was conducted using the products of the first round PCR as templates (Figure 5A and Figure S3). These data demonstrated the difference in constitutive expression of these nine *BnAGO4, BnRDR1*, and *BnDCL1* genes in *B. napus* leaves. Real-time quantitative PCR (RT-qPCR) was further used to gain insight into expression patterns of these genes in response to *S. sclerotiorum* inoculation at 0, 8, and 16 hpi. Interestingly, all of these genes, except *BnRDR1A, BnRDR1C1*, and *BnDCL1A*, were down-regulated to various degrees by *S. sclerotiorum* inoculation. Notably, 4 *AGO4s* were all markedly reduced by 2.2~10.2 folds at 16 hpi (Figure 5B). This result suggested the possible involvement of these genes in the interactions between *B. napus* and *S. sclerotiorum*.

All these genes with the exception of 2 *BnRDR1* (*BnRDR1A* and *BnRDR1C1*), carried CGCG elements in the DNA sequences upstream of their coding regions (Figure 4) and we conjectured that the lowered expression of these genes might have resulted from up-regulation of *CAMTA* genes in response to *S. sclerotiorum* inoculation. To test this hypothesis, we examined the expression of *BnCAMTA3* genes after inoculation with this pathogen. *BnCAMTA3* genes were selected since *AtCAMTA3* has been reported to function in regulating plant defense (Du et al., 2009; Nie et al., 2012; Rahman et al., 2016). Four *BnCAMTA3* genes were identified through BLASTp searches against *B. napus* genome database using AtCAMTA3 as query. RT-qPCR assays demonstrated that expression of two out of four *BnCAMTA3* genes (*BnCAMTA3a* and *BnCAMTA3b*) increased strongly by 14.7 and 10.4 folds, respectively, at 8 h after *S. sclerotiorum* inoculation (Figure 5C). Given that CAMTA3 negatively regulates plant disease resistance through direct binding and subsequent repression of target defense-related genes (Du et al., 2009; Nie et al., 2012), our results indicate that RNA silencing might be regulated by CAMTAs during *B. napus–S. sclerotiorum* interactions.

### Arabidopsis *dcl, ago*, and *rdr* mutants commonly exhibited altered susceptibility to *S. sclerotiorum*

**Figure 6:**
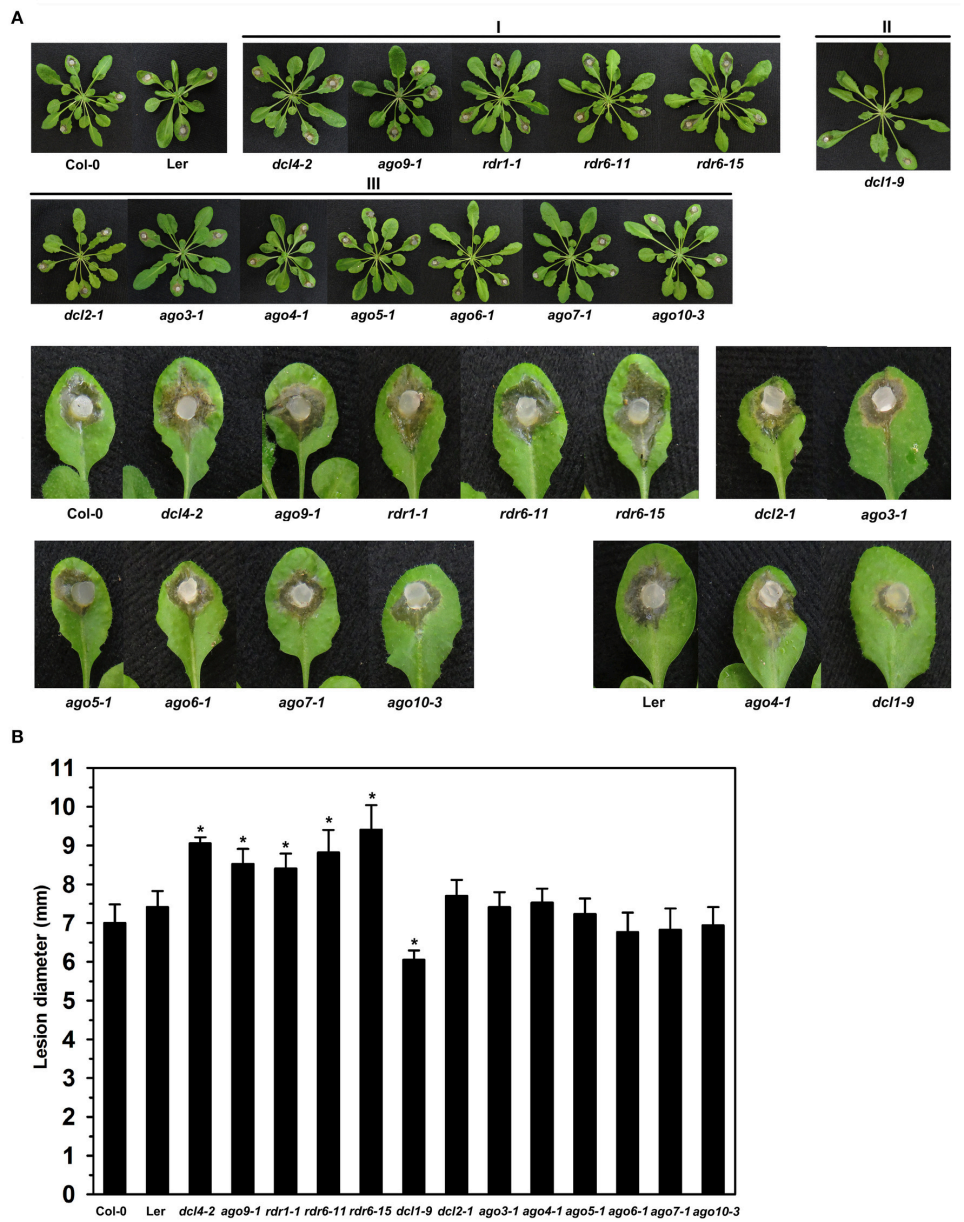
**Susceptibility to *S. sclerotiorum* in Arabidopsis *dcl*, *ago*, and *rdr* mutants. (A)** Disease symptoms of *dcl, ago*, and *rdr* mutants and wild type plants after inoculation with *S. sclerotiorum*. Photographs were taken at 24 hpi. I, mutants exhibiting enhanced susceptibility; II, mutants displaying enhanced resistance; and III, mutants showing similar disease phenotypes to wild-type plants. **(B)** Statistical analysis of disease severity. The inoculation analysis was performed three times, each in at least 10 plants for all mutants. The lesion size data were statistically analyzed using SPSS software. Significance of the differences between mean values was determined with Student's *t*-test. Error bars indicate *SD*, while asterisks (^*^) indicate significant difference at *p* < 0.05.

The observation in this study that expression of *BnDCL1, BnAGO4*, and *BnRDR1* genes altered significantly in response to *S. sclerotiorum* inoculation and our previous findings that Arabidopsis *ago1* and *ago2* mutant plants exhibit enhanced susceptibility to *S. sclerotiorum* (Cao et al., 2016) prompted us to assess the possible role of DCL, AGO, and RDR-mediated RNA silencing in plant resistance to this pathogen. A total of 13 *A. thaliana dcl, ago*, and *rdr* mutants were tested. They included three *dcl*, seven *ago*, and three *rdr* mutants, all in Col-0 background except *dcl1-9* and *ago4-1* which are derived from Ler ecotype. Based on the phenotypes after *S. sclerotiorum* inoculation, the mutants could be divided into three classes. Class I comprised those exhibiting enhanced susceptibility, Class II contained mutants displaying enhanced resistance, while Class III included mutants showing similar disease phenotypes to wild-type plants (Figure 6). The mutants *dcl4-2, ago9-1, rdr1-1, rdr6-11*, and *rdr6-15* were more susceptible to *S. sclerotiorum* challenge by showing more severe necrosis and larger size of disease lesions than wild-type plants (Figure 6). By contrast, the mutant *dcl1-9* was found to be more resistant since it displayed less necrosis and smaller size of disease lesions when compared with Ler plants upon *S. sclerotiorum* inoculation (Figure 6). However, the *dcl2-1, ago3-1, ago4-1, ago5-1, ago6-1, ago7-1*, and *ago10-3* mutant plants exhibited similar disease susceptibility as the wild-type plants with respect to severity of necrosis and size of disease lesions (Figure 6). These data demonstrate the involvement of DCL, AGO, and RDR-mediated RNA silencing in plant resistance to the necrotrophic fungal pathogen *S. sclerotiorum*.

## Discussion

RNA silencing is a versatile molecular mechanism to regulate diverse biological processes through altering transcript accumulation of genes essential to these processes. In this study, we identified three gene families encoding key components of RNA silencing machinery in *B. napus* and its progenitors *B. rapa* and *B. oleracea*. We demonstrated the important role of RNA silencing in plant resistance to the fungal pathogen *S. sclerotiorum* and indicated a possible new molecular mechanism to regulate RNA silencing in response to this pathogen. Our results provide insights into composition, function, and mechanism of RNA silencing in plants.

### RNA silencing machinery in *B. napus*

DCL, AGO, and RDR are key components of plant RNA silencing machinery. Our results demonstrate that *B. napus* possesses 8 *BnDCL*, 27 *BnAGO*, and 16 *BnRDR* genes. This gene copy numbers are much larger than those in *A. thaliana* although the two species belong to the same family (Brassicaceae). However, considering that *B. napus* is tetraploid while *A. thaliana* is diploid, it is clear that each haploidy in the two species carries the same number (4) of DCL genes but different number of AGO and RDR genes. The haploid *B. napus* contains more AGO and RDR genes than *A. thaliana*. *B. napus* is an allotetraploid from crossing between *B. oleracea* and *B. rapa*, followed by chromosome doubling (Chalhoub et al., 2014). Our results demonstrate that *B. napus* genome includes almost all copies of these RNA silencing machinery genes from its progenitor species *B. rapa* and *B. oleracea*. In addition, *B. napus* genome contains three extra copies of *RDR5* genes, indicating that the *RDR5* group in *B. napus* appears to have undergone further expansion through duplication during evolution. Moreover, compared with *A. thaliana*, some *AGO* and *RDR* genes such as *AGO1, AGO4, AGO9*, and *RDR5* have significantly expanded in these *Brassica* species. The *B. napus* subgenomes A and C bear more *RDR5* genes but similar *AGO* genes compared with genomes of its progenitor species *B. rapa* and *B. oleracea*, suggesting that the expansion of *RDR5* genes is likely to occur after *B. napus* formation while that of *AGO1, AGO4*, and *AGO9* may have occurred before *B. napus* formation. Additionally, members of *AGO7, AGO8*, and *AGO9* as well as *RDR4* are unevenly distributed in the A and C subgenomes of *B. napus*. This asymmetric distribution may have resulted from homeologous exchanges, which is also most likely the source of further expansion of these two families and it is consistent with the previous report (Chalhoub et al., 2014). Considering that *B. napus* genome contains at least two copies of each *DCL, AGO*, and *RDR* genes except *AGO8*, and that all four members of BnAGO4 are expressed constitutively and/or in response to pathogen inoculation (Figure 5) and are thus most probably functional, it is interesting to understand whether these genes functions redundantly or differentially.

Variation in composition of RNA machinery, especially composition of different gene groups (DCL1, DCL2, DCL3, and DCL4 for DCL; AGO2/3/7, AGO1/5/10, and AGO4/6/8/9 for AGO as well as RDR1/2/6 and RDR3/4/5 for RDR) seems to widely exist in plants. For example, the tomato genome contains four DCL2 but only a single ortholog of the other DCLs (Bai et al., 2012). Similarly, tomato AGO1/5/10 group consists of two AGO1 and AGO10 each, AGO2/3/7 group comprises two AGO2, AGO4/6/8/9 group is constituted of four AGO4s, and a new AGO member, but lacks AGO8 and AGO9 (Bai et al., 2012). Another species of the Solanaceae family, *N. benthamiana*, has identical number of AGO1/5/10 group genes, a similar number of AGO4/6/8/9 group genes but different number of AGO2/3/7 group genes when compared with tomato. The *N. benthamiana* AGO4/6/8/9 group bears two AGO4s, while the AGO2/3/7 group lacks AGO3 (Nakasugi et al., 2013). In addition, grape AGO1/5/10 group consists of two AGO10s, while AGO2/3/7 group includes two AGO2s (Zhao et al., 2015). Regarding RDR, tomato RDR1/2/6 group consists of two RDR6s, while this group in grape contains two RDR1s. The *N. benthamiana* genome apparently does not carry any RDR3/4/5 group genes, while tomato and grape RDR3/4/5 group both lack RDR4 and RDR5 but contain two and one RDR3, respectively (Bai et al., 2012; Nakasugi et al., 2013; Zhao et al., 2015).

### Function of RNA silencing in plant resistance to fungal pathogens

It is well-known that RNA silencing can protect plants from viral infection (Ding and Voinnet, 2007; Llave, 2010; Incarbone and Dunoyer, 2013; Wu et al., 2015). This antiviral immunity involves production of virus-derived small interfering RNAs (viRNAs) and results in specific silencing of viruses by viRNA-guided effector complexes. Apart from defense against viruses, RNA silencing also plays a role in plant defense against bacterial pathogens (Katiyar-Agarwal et al., 2006; Navarro et al., 2006; Voinnet, 2008; Robert-Seilaniantz et al., 2011). Similar to viruses, bacteria have also developed mechanisms to suppress RNA silencing in order to infect successfully (Navarro et al., 2008). More recently, functions of RNA silencing in plant resistance to fungal pathogens are being revealed. Plant miRNAs are differentially expressed in response to inoculation with fungal pathogens, such as *Erysiphe graminis* (Xin et al., 2010), *Fusarium virguliforme* (Radwan et al., 2011), *V. dahliae* (Yin et al., 2012; Yang et al., 2013), *V. longisporum* (Shen et al., 2014), *M. oryzae* (Li et al., 2014), and *B. cinerea* (Jin and Wu, 2015). More importantly, mutants of key components of the RNA silencing machinery exhibit altered susceptibility to fungal pathogens including two species of *Verticillium* (Ellendorff et al., 2009; Shen et al., 2014). Moreover, it has been reported that *B. cinerea* produces small RNAs to suppress plant defense by hijacking host RNA interference pathways (Weiberg et al., 2013). It is interesting to elucidate the function and mechanism of RNA silencing in the interactions between the important oil crop *B. napus* and the devastating fungal pathogen *S. sclerotiorum*. Previously, we have identified the miRNAs involved in this plant-pathogen interaction, many of which targeting genes involved in plant defense. Besides, three miRNAs (ath-miR168a_1ss21AC, aly-miR403a-3p_L+1, and bna-miR403) target AGO1 and AGO2 which are two key components of RNA silencing. We further found that Arabidopsis *ago1-27, ago1-33*, and *ago2-1* mutant plants exhibit enhanced susceptibility to *S. sclerotiorum* (Cao et al., 2016), thus providing a clue to the important roles of RNA silencing in the interactions between *B. napus* and *S. sclerotiorum*. In this study, we identified gene families encoding DCL, AGO, and RDR, three key components of RNA silencing in *B. napus*. We found that these genes, represented by all members of three groups of genes (4 *BnAGO4*, 3 *BnRDR1*, and 2 *BnDCL1* genes), are differentially expressed in response to *S. sclerotiorum* inoculation (Figure 5B). Our results showed that the expression divergence was present among members belonging to the same gene group after *S. sclerotiorum* inoculation (Figure 5B), which suggested that gene members within the same groups may have diverse functions in this plant-pathogen interaction. AtAGO4 is one of the critical components in the transcriptional gene-silencing pathway associated with siRNA that directs DNA methylation (Zilberman et al., 2004; Qi et al., 2006) and is required for resistance to *P. syringae* (Agorio and Vera, 2007). AtRDR1 is elicited by salicylic acid (SA) treatment and viral infection (Yu et al., 2003; Diaz-Pendon et al., 2007; Qi et al., 2009). Together, these results suggest that AGO4 and RDR1 may also contribute to *B. napus* defense against *S. sclerotiorum*. Furthermore, we assessed susceptibility toward *S. sclerotiorum* in 13 *dcl, ago*, and *rdr A. thaliana* mutants. As many as six *dcl, ago*, and *rdr* mutants exhibit altered susceptibility (Figure 6). These mutants include *dcl4-2, ago9-1, rdr1-1, rdr6-11, rdr6-15*, and *dcl1-9*. The mutated genes encode different RNA-silencing components. This is similar to what was found in the study involving another fungal pathogen *V. dahliae* (Ellendorff et al., 2009). Collectively, these results indicate that RNA silencing may be involved in interactions between plants and fungal pathogens. However, how these DCLs, AGOs, and RDRs regulate resistance to *S. sclerotiorum* is unclear. Recently, it has been reported that rice AGO18 functions through regulating AGO1 accumulation by direct binding with the AGO1-targeted miRNA (miR168; Wu et al., 2015). Whether a similar mechanism exists for AGOs or even RDRs and DCLs in Arabidopsis and *B. napus* is worthy of further study.

### Regulation of RNA silencing machiner*y*

A remaining challenge for RNA silencing is to dissect the mechanism through which the RNA silencing pathway itself can be regulated. Up to now, little of this mechanism has been uncovered except the evidence that the expression of components of the RNA silencing pathway is subject to negative feedback regulation by their own miRNA products. For example, miR162 targets DCL1, miR168 targets AGO1, and miR403 targets AGO2. However, there is no more information regarding regulatory mechanisms for the other RNA silencing components. Considering the importance of RNA silencing pathway, we are curious to know whether other mechanisms exist that confer additional layers of regulation on the RNA silencing machinery. Intriguingly, in present study, we found that substantial number of *B. napus* DCL, AGO, and RDR genes (21 out of a total of 51) possessed CAMTA/SR binding sites [(A/C/G) CGCG (C/G/T)] in their promoter sequences (Figure 4 and Table S4). CAMTAs, especially CAMTA3, contribute to plant defense responses by direct binding and thereby regulating the expression of the target genes (Yang and Poovaiah, 2002; Du et al., 2009; Nie et al., 2012). Therefore, we suspect that the expression of these CGCG-element-containing RNA silencing components may be regulated by CAMTAs. To confirm this possibility, we examined the expression of CAMTA genes and these CGCG-element-containing RNA silencing genes in *B. napus* in response to *S. sclerotiorum* inoculation. The results of this expression analysis indicate that *S. sclerotiorum* inoculation strongly induced the expression of *BnCAMTA3* genes while it significantly suppressed that of many CGCG-element-containing *BnAGO, BnDCL* and *BnRDR* genes (Figure 5). Moreover, another work in our laboratory has revealed that *Atcamta3* mutant plants exhibit enhanced resistance to *S. sclerotiorum* (Rahman et al., unpublished data). Taken together, our results suggest that RNA silencing might be regulated by CAMTA3. Nevertheless, further confirmation of CAMTA binding activity with CGCG-element-containing RNA silencing genes by other assays such as EMSA and its effect on expression of these genes will provide more straightforward evidence to support this intriguing likely mechanism for regulation of RNA silencing machinery.

It is well-known that the functions of CAMTAs are dependent on their interaction with Ca^2+^/CaM (Choi et al., 2005; Du et al., 2009) and these genes, especially *CAMTA3*, are widely involved in plant defense (Yang and Poovaiah, 2002; Du et al., 2009; Nie et al., 2012; Rahman et al., 2016). For instance, knockout of *AtCAMTA3* leads to increased accumulation of salicylic acid and enhanced host disease resistance to both bacterial (Du et al., 2009) and fungal pathogens (Nie et al., 2012) and nonhost resistance to bacterial pathogen (Rahman et al., 2016) but reduced resistance against insect herbivores (Qiu et al., 2012). Similarly, one rice CAMTA mutant (*oscbt-1*) exhibits enhanced resistance to blast fungal pathogen and leaf blight bacterial pathogen (Koo et al., 2009). Given that expression of some RNA silencing machinery may be regulated by CAMATs and both of CAMTA as well as RNA silencing contribute to plant resistance, it will be intriguing to explore whether the role of CAMTAs in regulating plant defense response can be attributed to its regulation of RNA silencing and thus opening a possibility to link Ca^2+^ signaling and RNA silencing together to provide a novel facet of Ca^2+^ signaling in regulation of plant disease resistance.

## Conclusions

The *B. napus* genome possessed 8 *Dicer-like* (*DCL*), 27 *Argonaute* and 16 *RNA-dependent RNA polymerase* (*RDR*) genes. The *B. napus* genome expanded *RDR5* genes compared with that of its progenitors *B. oleracea* and *B. rapa* and all genomes of three *Brassica* species expanded *AGO1, AGO4*, and *AGO9* genes compared with the Arabidopsis genome. *B. napus DCL, AGO*, and *RDR* genes widely (21 out of 51) harbored a CAMTA-binding site (CGCG box) in their promoter regions. Inoculation with *S. sclerotiorum* strongly induced the expression of *BnCAMTA3* genes while significantly reduced that of many CGCG-containing RNA silencing component genes. Our results suggested that RNA silencing machinery might be targeted by CAMTA3. Furthermore, mutant analyses demonstrated that six out of 13 *dcl, ago*, and *rdr* mutants exhibited altered susceptibility to *S. sclerotiorum*. These results indicate the important role of RNA silencing in plant resistance to this devastating fungal pathogen.

## Author note

During the review process of this manuscript, a paper on bioinformatics identification of *DCL, AGO*, and *RDR* families in *Brassica napus* was accepted for publication (Zhao et al., 2016).

## Author contributions

The project was coordinated by X-ZC. J-YC, and Y-PX conducted the bioinformatics and phylogenetic analyses. J-YC, Y-PX, and HR carried out the gene expression assays. J-YC, WL, and S-SL performed disease resistance evaluation analyses. J-YC designed and performed the statistical analysis. X-ZC conceived of the study, and participated in its design and coordination. X-ZC and J-YC prepared the manuscript. All authors read and approved the final manuscript.

### Conflict of interest statement

The authors declare that the research was conducted in the absence of any commercial or financial relationships that could be construed as a potential conflict of interest.

## References

[B1] Agorio A. Vera P. (2007). ARGONAUTE4 is required for resistance to *Pseudomonas syringae* in Arabidopsis. Plant Cell 19, 3778–3790. 10.1105/tpc.107.054494 17993621 PMC2174867

[B2] Bai M. Yang G. S. Chen W. T. Mao Z. C. Kang H. X. Chen G. H.. (2012). Genome-wide identification of Dicer-like, Argonaute and RNA-dependent RNA polymerase gene families and their expression analyses in response to viral infection and abiotic stresses in *Solanum lycopersicum*. Gene 501, 52–62. 10.1016/j.gene.2012.02.009 22406496

[B3] Baulcombe D. (2004). RNA silencing in plants. Nature 431, 356–363. 10.1038/nature02874 15372043

[B4] Bjellqvist B. Hughes G. J. Pasquali C. Paquet N. Ravier F. Sanchez J. C.. (1993). The focusing positions of polypeptides in immobilized pH gradients can be predicted from their amino acid sequences. Electrophoresis 14, 1023–1031. 10.1002/elps.11501401163 8125050

[B5] Bologna N. G. Voinnet O. (2014). The diversity, biogenesis, and activities of endogenous silencing small RNAs in Arabidopsis. Annu. Rev. Plant Biol. 65, 473–503. 10.1146/annurev-arplant-050213-035728 24579988

[B6] Cao J. Y. Xu Y. P. Zhao L. Li S. S. Cai X. Z. (2016). Tight regulation of the interaction between *Brassica napus* and *Sclerotinia sclerotiorum* at the microRNA level. Plant Mol. Biol. 92, 39–55. 10.1007/s11103-016-0494-3 27325118

[B7] Carmell M. A. Hannon G. J. (2004). RNase III enzymes and the initiation of gene silencing. Nat. Struct. Mol. Biol. 11, 214–218. 10.1038/nsmb729 14983173

[B8] Chalhoub B. Denoeud F. Liu S. Parkin I. A. P. Tang H. Wang X.. (2014). Early allopolyploid evolution in the post-neolithic *Brassica napus* oilseed genome. Science 345, 950–953. 10.1126/science.1253435 25146293

[B9] Choi M. S. Kim M. C. Yoo J. H. Moon B. C. Koo S. C. Park B. O.. (2005). Isolation of a calmodulin-binding transcription factor from rice (*Oryza sativa* L.). J. Biol. Chem. 280, 40820–40831. 10.1074/jbc.M504616200 16192280

[B10] Csorba T. Kontra L. Burgyán J. (2015). Viral silencing suppressors: tools forged to fine-tune host-pathogen coexistence. Virology 479, 85–103. 10.1016/j.virol.2015.02.028 25766638

[B11] Deleris A. Gallego-Bartolome J. Bao J. Kasschau K. D. Carrington J. C. Voinnet O. (2006). Hierarchical action and inhibition of plant Dicer-like proteins in antiviral defense. Science 313, 68–71. 10.1126/science.1128214 16741077

[B12] Diaz-Pendon J. A. Li F. Li W. X. Ding S. W. (2007). Suppression of antiviral silencing by cucumber mosaic virus 2b protein in Arabidopsis is associated with drastically reduced accumulation of three classes of viral small interfering RNAs. Plant Cell 19, 2053–2063. 10.1105/tpc.106.047449 17586651 PMC1955711

[B13] Ding S. W. Voinnet O. (2007). Antiviral immunity directed by small RNAs. Cell 130, 413–426. 10.1016/j.cell.2007.07.039 17693253 PMC2703654

[B14] Du L. Ali G. S. Simons K. A. Hou J. Yang T. Reddy A. S. N. (2009). Ca^2+^/calmodulin regulates salicylic-acid-mediated plant immunity. Nature 457, 1154–1158. 10.1038/nature07612 19122675

[B15] Edgar R. C. (2004). MUSCLE: multiple sequence alignment with high accuracy and high throughput. Nucleic Acids Res. 32, 1792–1797. 10.1093/nar/gkh340 15034147 PMC390337

[B16] Ellendorff U. Fradin E. F. de Jonge R. Thomma B. P. H. J. (2009). RNA silencing is required for Arabidopsis defence against Verticillium wilt disease. J. Exp. Bot. 60, 591–602. 10.1093/jxb/ern306 19098131 PMC2651451

[B17] Hannon G. J. (2002). RNA interference. Nature 418, 244–251. 10.1038/418244a 12110901

[B18] Higo K. Ugawa Y. Iwamoto M. Korenaga T. (1999). Plant *cis*-acting regulatory DNA elements (PLACE) database. Nucleic Acids Res. 27, 297–300. 10.1093/nar/27.1.297 9847208 PMC148163

[B19] Hutvagner G. Simard M. J. (2008). Argonaute proteins: key players in RNA silencing. Nat. Rev. Mol. Cell Biol. 9, 22–32. 10.1038/nrm2321 18073770

[B20] Incarbone M. Dunoyer P. (2013). RNA silencing and its suppression: novel insights from in planta analyses. Trends Plant Sci. 18, 382–392. 10.1016/j.tplants.2013.04.001 23684690

[B21] Jaubert M. Bhattacharjee S. Mello A. F. S. Perry K. L. Moffett P. (2011). ARGONAUTE2 mediates RNA-silencing antiviral defenses against *Potato virus X* in Arabidopsis. Plant Physiol. 156, 1556–1564. 10.1104/pp.111.178012 21576511 PMC3135937

[B22] Jin W. Wu F. (2015). Characterization of miRNAs associated with *Botrytis cinerea* infection of tomato leaves. BMC Plant Biol. 15:1. 10.1186/s12870-014-0410-4 25592487 PMC4311480

[B23] Kapoor M. Arora R. Lama T. Nijhawan A. Khurana J. P. Tyagi A. K.. (2008). Genome-wide identification, organization and phylogenetic analysis of Dicer-like, Argonaute and RNA-dependent RNA Polymerase gene families and their expression analysis during reproductive development and stress in rice. BMC Genomics 9:451. 10.1186/1471-2164-9-451 18826656 PMC2576257

[B24] Katiyar-Agarwal S. Morgan R. Dahlbeck D. Borsani O. Villegas A. Jr. Zhu J. K.. (2006). A pathogen-inducible endogenous siRNA in plant immunity. Proc. Natl. Acad. Sci. U.S.A. 103, 18002–18007. 10.1073/pnas.0608258103 17071740 PMC1693862

[B25] Koo S. C. Choi M. S. Chun H. J. Shin D. B. Park B. S. Kim Y. H.. (2009). The calmodulin-binding transcription factor OsCBT suppresses defense responses to pathogens in rice. Mol. Cells 27, 563–570. 10.1007/s10059-009-0081-4 19466605

[B26] Li Y. Lu Y. G. Shi Y. Wu L. Xu Y. J. Huang F.. (2014). Multiple rice microRNAs are involved in immunity against the blast fungus *Magnaporthe oryzae*. Plant Physiol. 164, 1077–1092. 10.1104/pp.113.230052 24335508 PMC3912081

[B27] Liu Q. Feng Y. Zhu Z. (2009). Dicer-like (DCL) proteins in plants. Funct. Integr. Genomics 9, 277–286. 10.1007/s10142-009-0111-5 19221817

[B28] Llave C. (2010). Virus-derived small interfering RNAs at the core of plant-virus interactions. Trends Plant Sci. 15, 701–707. 10.1016/j.tplants.2010.09.001 20926332

[B29] Lynn K. Fernandez A. Aida M. Sedbrook J. Tasaka M. Masson P.. (1999). The PINHEAD/ZWILLE gene acts pleiotropically in Arabidopsis development and has overlapping functions with the ARGONAUTE1 gene. Development 126, 469–481. 9876176 10.1242/dev.126.3.469

[B30] Margis R. Fusaro A. F. Smith N. A. Curtin S. J. Watson J. M. Finnegan E. J.. (2006). The evolution and diversification of Dicers in plants. FEBS Lett. 580, 2442–2450. 10.1016/j.febslet.2006.03.072 16638569

[B31] Moazed D. (2009). Small RNAs in transcriptional gene silencing and genome defence. Nature 457, 413–420. 10.1038/nature07756 19158787 PMC3246369

[B32] Moissiard G. Parizotto E. A. Himber C. Voinnet O. (2007). Transitivity in Arabidopsis can be primed, requires the redundant action of the antiviral Dicer-like 4 and Dicer-like 2, and is compromised by viral-encoded suppressor proteins. RNA 13, 1268–1278. 10.1261/rna.541307 17592042 PMC1924903

[B33] Moissiard G. Voinnet O. (2006). RNA silencing of host transcripts by cauliflower mosaic virus requires coordinated action of the four Arabidopsis Dicer-like proteins. Proc. Natl. Acad. Sci. U.S.A. 103, 19593–19598. 10.1073/pnas.0604627103 17164336 PMC1698440

[B34] Nakasugi K. Crowhurst R. N. Bally J. Wood C. C. Hellens R. P. Waterhouse P. M. (2013). *De novo* transcriptome sequence assembly and analysis of RNA silencing genes of *Nicotiana benthamiana*. PLoS ONE 8:e59534. 10.1371/journal.pone.0059534 23555698 PMC3610648

[B35] Navarro L. Dunoyer P. Jay F. Arnold B. Dharmasiri N. Estelle M.. (2006). A plant miRNA contributes to antibacterial resistance by repressing auxin signaling. Science 312, 436–439. 10.1126/science.1126088 16627744

[B36] Navarro L. Jay F. Nomura K. He S. Y. Voinnet O. (2008). Suppression of the microRNA pathway by bacterial effector proteins. Science 321, 964–967. 10.1126/science.1159505 18703740 PMC2570098

[B37] Nie H. Zhao C. Wu G. Wu Y. Chen Y. Tang D. (2012). SR1, a calmodulin-binding transcription factor, modulates plant defense and ethylene-induced senescence by directly regulating NDR1 and EIN3. Plant Physiol. 158, 1847–1859. 10.1104/pp.111.192310 22345509 PMC3320190

[B38] Parent J. S. Bouteiller N. Elmayan T. Vaucheret H. (2015). Respective contributions of Arabidopsis DCL2 and DCL4 to RNA silencing. Plant J. 81, 223–232. 10.1111/tpj.12720 25376953

[B39] Qi X. Bao F. S. Xie Z. (2009). Small RNA deep sequencing reveals role for *Arabidopsis thaliana* RNA-dependent RNA polymerases in viral siRNA biogenesis. PLoS ONE 4:e4971. 10.1371/journal.pone.0004971 19308254 PMC2654919

[B40] Qi Y. He X. Wang X. J. Kohany O. Jurka J. Hannon G. J. (2006). Distinct catalytic and non-catalytic roles of ARGONAUTE4 in RNA-directed DNA methylation. Nature 443, 1008–1012. 10.1038/nature05198 16998468

[B41] Qian Y. Cheng Y. Cheng X. Jiang H. Zhu S. Cheng B. (2011). Identification and characterization of Dicer-like, Argonaute and RNA-dependent RNA polymerase gene families in maize. Plant Cell Rep. 30, 1347–1363. 10.1007/s00299-011-1046-6 21404010

[B42] Qiu Y. Xi J. Du L. Suttle J. C. Poovaiah B. W. (2012). Coupling calcium/calmodulin-mediated signaling and herbivore-induced plant response through calmodulin-binding transcription factor AtSR1/CAMTA3. Plant Mol. Biol. 79, 89–99. 10.1007/s11103-012-9896-z 22371088

[B43] Radwan O. Liu Y. Clough S. J. (2011). Transcriptional analysis of soybean root response to *Fusarium virguliforme*, the causal agent of sudden death syndrome. Mol. Plant Microb. Interact. 24, 958–972. 10.1094/MPMI-11-10-0271 21751852

[B44] Rahman H. Yang J. Xu Y. P. Munyampundu J. P. Cai X. Z. (2016). Phylogeny of plant CAMTAs and role of AtCAMTAs in nonhost resistance to *Xanthomonas oryzae* pv. oryzae. Front. Plant Sci. 7:117. 10.3389/fpls.2016.00177 26973658 PMC4770041

[B45] Robert-Seilaniantz A. MacLean D. Jikumaru Y. Hill L. Yamaguchi S. Kamiya Y.. (2011). The microRNA miR393 re-directs secondary metabolite biosynthesis away from camalexin and towards glucosinolates. Plant J. 67, 218–231. 10.1111/j.1365-313X.2011.04591.x 21457368

[B46] Schiebel W. Pélissier T. Riedel L. Thalmeir S. Schiebel R. Kempe D.. (1998). Isolation of an RNA-Directed RNA polymerase-specific cDNA clone from tomato. Plant Cell 10, 2087–2101. 9836747 10.1105/tpc.10.12.2087 PMC143969

[B47] Shen D. Suhrkamp I. Wang Y. Liu S. Menkhaus J. Verreet J. A.. (2014). Identification and characterization of microRNAs in oilseed rape (*Brassica napus*) responsive to infection with the pathogenic fungus *Verticillium longisporum* using Brassica AA (*Brassica rapa*) and CC (*Brassica oleracea*) as reference genomes. New Phytol. 204, 577–594. 10.1111/nph.12934 25132374

[B48] Sijen T. Fleenor J. Simmer F. Thijssen K. L. Parrish S. Timmons L.. (2001). On the role of RNA amplification in dsRNA-triggered gene silencing. Cell 107, 465–476. 10.1016/S0092-8674(01)00576-1 11719187

[B49] Tamura K. Peterson D. Peterson N. Stecher G. Nei M. Kumar S. (2011). MEGA5: molecular evolutionary genetics analysis using maximum likelihood, evolutionary distance, and maximum parsimony methods. Mol. Biol. Evol. 28, 2731–2739. 10.1093/molbev/msr121 21546353 PMC3203626

[B50] Tijsterman M. Ketting R. F. Plasterk R. H. A. (2002). The genetics of RNA silencing. Annu. Rev. Genet. 36, 489–519. 10.1146/annurev.genet.36.043002.091619 12429701

[B51] Ullu E. Tschudi C. Chakraborty T. (2004). RNA interference in protozoan parasites. Cell. Microbiol. 6, 509–519. 10.1111/j.1462-5822.2004.00399.x 15104593

[B52] Voinnet O. (2008). Post-transcriptional RNA silencing in plant-microbe interactions: a touch of robustness and versatility. Curr. Opin. Plant Biol. 11, 464–470. 10.1016/j.pbi.2008.04.006 18583181

[B53] Weiberg A. Wang M. Lin F. M. Zhao H. Zhang Z. Kaloshian I.. (2013). Fungal small RNAs suppress plant immunity by hijacking host RNA interference pathways. Science 342, 118–123. 10.1126/science.1239705 24092744 PMC4096153

[B54] Wu J. Yang Z. Wang Y. Zheng L. Ye R. Ji Y.. (2015). Viral-inducible Argonaute18 confers broad-spectrum virus resistance in rice by sequestering a host microRNA. Elife 4:e05733. 10.7554/eLife.05733 25688565 PMC4358150

[B55] Xie Z. Johansen L. K. Gustafson A. M. Kasschau K. D. Lellis A. D. Zilberman D.. (2004). Genetic and functional diversification of small RNA pathways in plants. PLoS Biol. 2:E104. 10.1371/journal.pbio.0020104 15024409 PMC350667

[B56] Xin M. Wang Y. Yao Y. Xie C. Peng H. Ni Z.. (2010). Diverse set of microRNAs are responsive to powdery mildew infection and heat stress in wheat (*Triticum aestivum* L.). BMC Plant Biol. 10:123. 10.1186/1471-2229-10-123 20573268 PMC3095282

[B57] Yadav C. B. Muthamilarasan M. Pandey G. Prasad M. (2015). Identification, characterization and expression profiling of Dicer-Like, Argonaute and RNA-dependent RNA polymerase gene families in foxtail millet. Plant Mol. Biol. Rep. 33, 43–55. 10.1007/s11105-014-0736-y

[B58] Yang L. Jue D. Li W. Zhang R. Chen M. Yang Q. (2013). Identification of miRNA from eggplant (*Solanum melongena* L.) by small RNA deep sequencing and their response to *Verticillium dahliae* infection. PLoS ONE 8:e72840. 10.1371/journal.pone.0072840 24015279 PMC3754920

[B59] Yang T. B. Poovaiah B. W. (2002). A calmodulin-binding/CGCG box DNA-binding protein family involved in multiple signaling pathways in plants. J. Biol. Chem. 277, 45049–45058. 10.1074/jbc.M207941200 12218065

[B60] Yin Z. Li Y. Han X. Shen F. (2012). Genome-wide profiling of miRNAs and other small non-coding RNAs in the *Verticillium dahliae*-inoculated cotton roots. PLoS ONE 7:e35765. 10.1371/journal.pone.0035765 22558219 PMC3338460

[B61] Yoshikawa M. Peragine A. Park M. Y. Poethig R. S. (2005). A pathway for the biogenesis of trans-acting siRNAs in Arabidopsis. Genes Dev. 19, 2164–2175. 10.1101/gad.1352605 16131612 PMC1221887

[B62] Yu B. Wang H. (2010). Translational inhibition by microRNAs in plants. Prog. Mol. Subcel. Biol. 50, 41–57. 10.1007/978-3-642-03103-8_3 19841880

[B63] Yu D. Fan B. MacFarlane S. A. Chen Z. (2003). Analysis of the involvement of an inducible Arabidopsis RNA-dependent RNA polymerase in antiviral defense. Mol. Plant Microb. Interact. 16, 206–216. 10.1094/MPMI.2003.16.3.206 12650452

[B64] Zhao H. Zhao K. Wang J. Chen X. Chen Z. Cai R. (2015). Comprehensive analysis of Dicer-Like, Argonaute, and RNA-dependent RNA polymerase gene families in grapevine (*Vitis Vinifer*a). J. Plant Growth Regul. 34, 108–121. 10.1007/s00344-014-9448-7

[B65] Zhao X. Zheng W. Zhong Z. Chen X. Wang A. Wang Z. (2016). Genome-wide analysis of RNA-interference pathway in *Brassica napus*, and the expression profile of BnAGOs in response to *Sclerotinia sclerotiorum* infection. Eur. J. Plant Pathol. 146, 565 10.1007/s10658-016-0942-6

[B66] Zhao Y. Liu W. Xu Y. P. Cao J. Y. Braam J. Cai X. Z. (2013). Genome-wide identification and functional analyses of calmodulin genes in Solanaceous species. BMC Plant Biol. 13:70. 10.1186/1471-2229-13-70 23621884 PMC3751459

[B67] Zhu H. Hu F. Wang R. Zhou X. Sze S. H. Liou L. W.. (2011). Arabidopsis Argonaute10 specifically sequesters miR166/165 to regulate shoot apical meristem development. Cell 145, 242–256. 10.1016/j.cell.2011.03.024 21496644 PMC4124879

[B68] Zilberman D. Cao X. Johansen L. K. Xie Z. Carrington J. C. Jacobsen S. E. (2004). Role of Arabidopsis ARGONAUTE4 in RNA-directed DNA methylation triggered by inverted repeats. Curr. Biol. 14, 1214–1220. 10.1016/j.cub.2004.06.055 15242620

