# Supplementary material for: Genome-Wide Identification of Dicer-Like, Argonaute, and RNA-Dependent RNA Polymerase Gene Families in Brassica Species and Functional Analyses of Their Arabidopsis Homologs in Resistance to Sclerotinia sclerotiorum
Source: Front Plant Sci. 2016 Oct 27;7:1614. doi: 10.3389/fpls.2016.01614 (PMC5081487; doi:10.3389/fpls.2016.01614)
Supplement: Supplementary file 5 [file Image1.PDF]

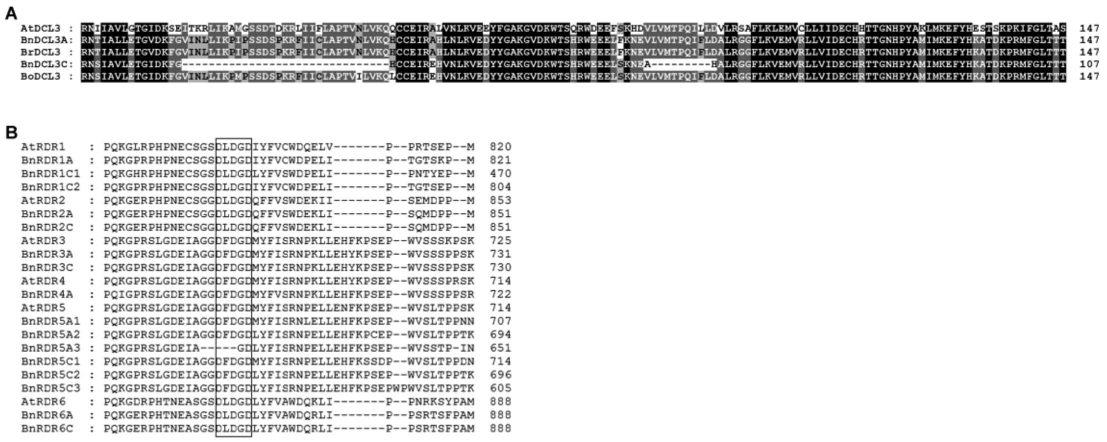

**Figure S1.** Alignment of DEAD domains of DCL3 proteins (A) and the catalytic regions in RdRP domains of RDR proteins (B) from *A. thaliana*, *B. rapa*, *B. oleracea* and *B. napus*. The conserved DLDGD and DFDGD motifs were black boxed. The protein sequences were aligned using MEGA 5.0. A "-" represents an amino acid deletion in the corresponding sequence. The positions corresponding to each protein are indicated at the end of each line.
