# Supplementary material for: Genome-Wide Identification of Dicer-Like, Argonaute, and RNA-Dependent RNA Polymerase Gene Families in Brassica Species and Functional Analyses of Their Arabidopsis Homologs in Resistance to Sclerotinia sclerotiorum
Source: Front Plant Sci. 2016 Oct 27;7:1614. doi: 10.3389/fpls.2016.01614 (PMC5081487; doi:10.3389/fpls.2016.01614)
Supplement: Supplementary file 6 [file Image2.PDF]

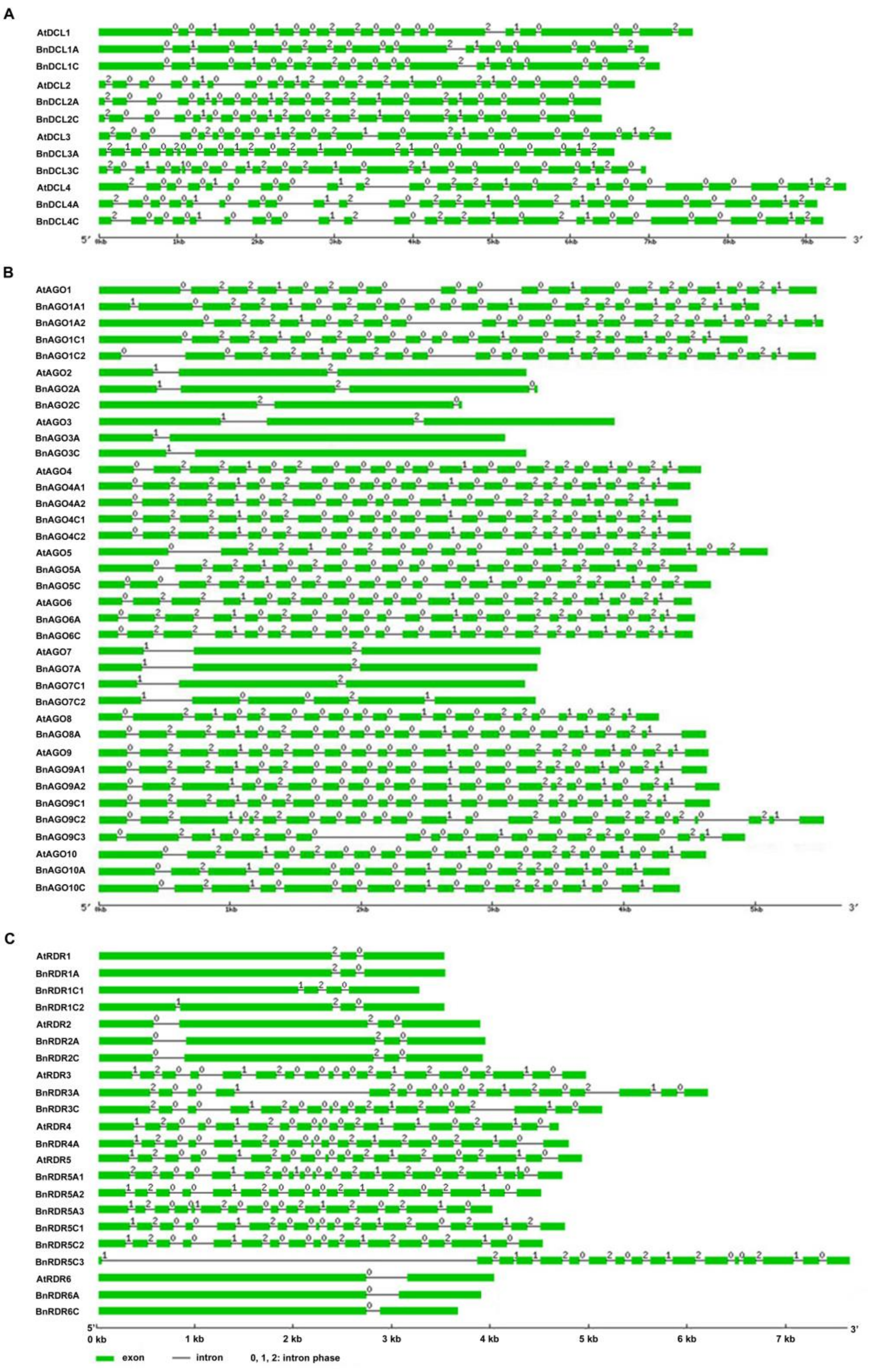

Figure S2. Exon-intron structures of *B. napus* and *A. thaliana DCL*, *AGO* and *RDR* genes. Exons (green boxes), introns (black lines) and intron phases (0, 1, 2) are shown. The gene structures were predicted using the online program GSDS1.0 (http://gsds.cbi.pku.edu.cn/) by comparing their full-length coding sequences (CDS) with their corresponding genomic sequences.
