# Supplementary material for: Genome-Wide Identification of Dicer-Like, Argonaute, and RNA-Dependent RNA Polymerase Gene Families in Brassica Species and Functional Analyses of Their Arabidopsis Homologs in Resistance to Sclerotinia sclerotiorum
Source: Front Plant Sci. 2016 Oct 27;7:1614. doi: 10.3389/fpls.2016.01614 (PMC5081487; doi:10.3389/fpls.2016.01614)
Supplement: Supplementary file 7 [file Image3.PDF]

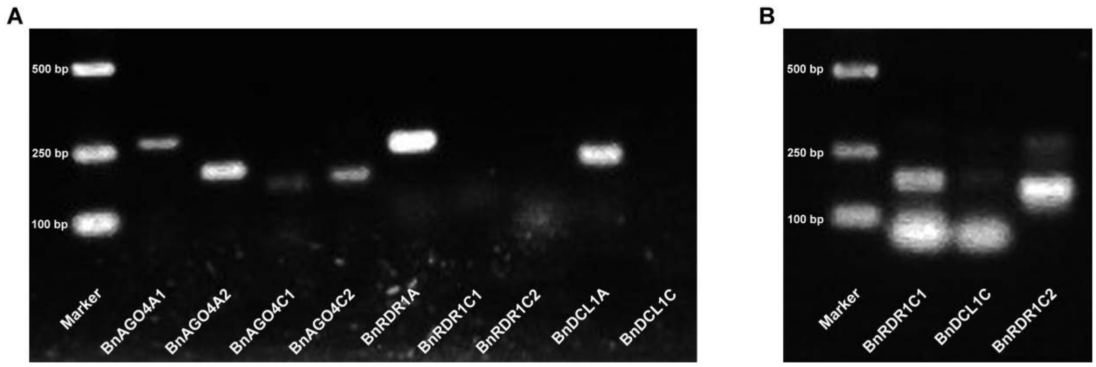

Figure S3. The original gel images for Figure 5A. Products of the first round (A) and second round (B) of PCRs were run in agrose gels. Size of DNA marker and genes for amplification are indicated.
